# A Novel Approach of Pericardial Suspension Method Combined with Tracheobronchial Stent Placement: A Life-Saving Case for Right Main Bronchial Stenosis

**DOI:** 10.70352/scrj.cr.25-0477

**Published:** 2026-01-31

**Authors:** Ryota Nagashima, Aki Fujiwara-Kuroda, Masato Aragaki, Kichizo Kaga, Tatsuya Kato, Naofumi Shinagawa

**Affiliations:** 1 Department of Thoracic Surgery, Hokkaido University Hospital, Sapporo, Hokkaido, Japan; 2 Department of Respiratory Medicine, Faculty of Medicine, Hokkaido University, Sapporo, Hokkaido, Japan

**Keywords:** bronchial stenosis, pericardial suspension, tracheobronchial stenting

## Abstract

**INTRODUCTION:**

There is no consensus regarding surgery and endotracheal treatment for bronchial stenosis. We report a case of right main bronchus stenosis treated using a novel approach of mediastinal mobilization with pericardial suspension.

**CASE PRESENTATION:**

A 57-year-old woman who developed radiation pneumonitis after postoperative radiation therapy for left breast cancer was admitted to our hospital due to worsening respiratory distress over 2 years. Chest CT revealed severe stenosis of the right main bronchus owing to lung destruction and scoliosis. Although isolated lung ventilation using a double-lumen tube was initiated, the ventilation was unstable. The patient underwent surgery, including sternal elevation using the Nuss technique, mediastinal mobilization using pericardial suspension, and tracheobronchial stenting to ameliorate bronchial stenosis.

**CONCLUSIONS:**

After surgery, oxygenation was no longer required. This novel pericardial suspension technique fundamentally corrects the extrinsic mediastinal shift, serving as an essential prerequisite for safe stenting against secondary tracheobronchomalacia in adult cases.

## Abbreviations

AT
: atrial tachycardia
PSVT
: paroxysmal supraventricular tachycardia
TBM
: tracheobronchomalacia
VV-ECMO
: veno-venous extracorporeal membrane oxygenation

## INTRODUCTION

No clear consensus exists on the treatment of bronchial stenosis; hence, determining the appropriate treatment approach can be challenging. We report a case of right bronchial stenosis caused by prolonged mediastinal displacement which was successfully treated with mediastinal mobilization surgery combined with tracheal/bronchial stenting. This case highlights the potential effectiveness of pericardial suspension as a mediastinal mobilization method for the treatment of bronchial stenosis.

## CASE PRESENTATION

**Fig. 1:**
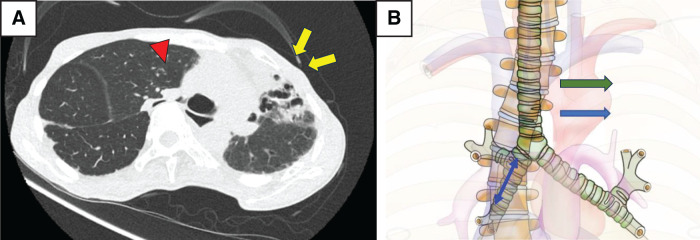
(**A**) Chest CT at initial examination: The left upper lobe shows features consistent with a destroyed lung (yellow arrows), the mediastinal organs are shifted to the left, and the right main bronchus is narrowed (red arrowhead). (**B**) Schematic drawing: Stenosis is observed in the area indicated by the blue double arrow. The mediastinum was mobilized to the left (green arrow) and lifted to the ventral side to open the endobronchial lumen.

A 57-year-old woman with a history of left breast cancer, bone metastases, and 34-degree Cobb angle scoliosis was admitted to our hospital with dyspnea. The patient underwent radiation therapy after mastectomy for left breast cancer. Two months later, she developed radiation pneumonitis. Upon admission, CT showed stenosis of the right main bronchus; isolated lung ventilation was performed owing to impaired consciousness, and dialysis was initiated for renal failure. However, due to unstable ventilation, the patient was transported to our hospital for treatment. Upon arrival, the patient was conscious and had a systolic blood pressure of 120–140 mmHg, heart rate of 60–80/min, and PaO_2_/FiO_2_ of 465. Chest CT image showed organizing pneumonia in the left upper lobe and scoliosis of the thoracic spine. These 2 factors caused a leftward deviation of the mediastinum and severe stenosis from the right main bronchus to the middle bronchial trunk (**Fig. 1**). Even after the transfer to our hospital, she exhibited unstable ventilation and required therapeutic intervention.

Bronchoscopic stenting was considered the initial treatment; however, a risk of stent migration existed due to enlargement of the tracheal bifurcation angle. In addition, compression between the stent and the vertebrae raised concerns regarding bronchial necrosis due to blood flow failure. Thus, it was contraindicated, and surgical mobilization of the mediastinum was considered. First, a Nuss sternotomy was performed to elevate the thorax anteriorly on day 6 of admission, and 2 Pectus bars (14 in, 12 mm wide) were inserted between the 3rd and 4th intercostal spaces. Postoperative bronchoscopy showed slight improvement in the right main bronchial stenosis; however, the ventilation volume remained unstable. To correct the mediastinal deviation, mediastinal mobilization with pericardial suspension was performed on admission day 9.

This procedure was initiated under VV-ECMO. The main settings for VV-ECMO were a pump speed of 3200 rpm and a blood flow of 3.5–3.8 L/min, with cannulation of the right femoral vein for drainage and the left femoral vein for return. After removing the 2 bars that had already been inserted, a median sternotomy was performed. The left brachiocephalic vein, superior vena cava, ascending aorta, and trachea were encircled, and the arch of the azygos vein was cut using a stapler. While the endobronchial lumen was observed using a bronchoscope, the mediastinal organs, including the trachea and ascending aorta, were pulled in several directions, including the right and ventral sides, with taping. However, lumen patency did not improve. Therefore, we made a median incision in the pericardium and elevated its right lateral edge anteriorly on the left side, and found that the best opening of the right main bronchus was achieved. Accordingly, the pericardium was lifted using the left anterior chest wall as a fulcrum. A nonabsorbable thread was placed over the right margin of the pericardium in a U shape and perforated across each of the 4 ribs. After closing the sternum, the perforated thread was sutured, with the knots buried subcutaneously in the anterior chest wall tissue to ensure permanent fixation, and the entire pericardium was completely lifted to the left ventral side. A single pectus bar was reinserted into the 4th intercostal space to prevent the sinking of the chest wall due to pericardial traction (**Supplementary Video**). The operative time was 311 min, and blood loss was 390 mL.

**Fig. 2:**
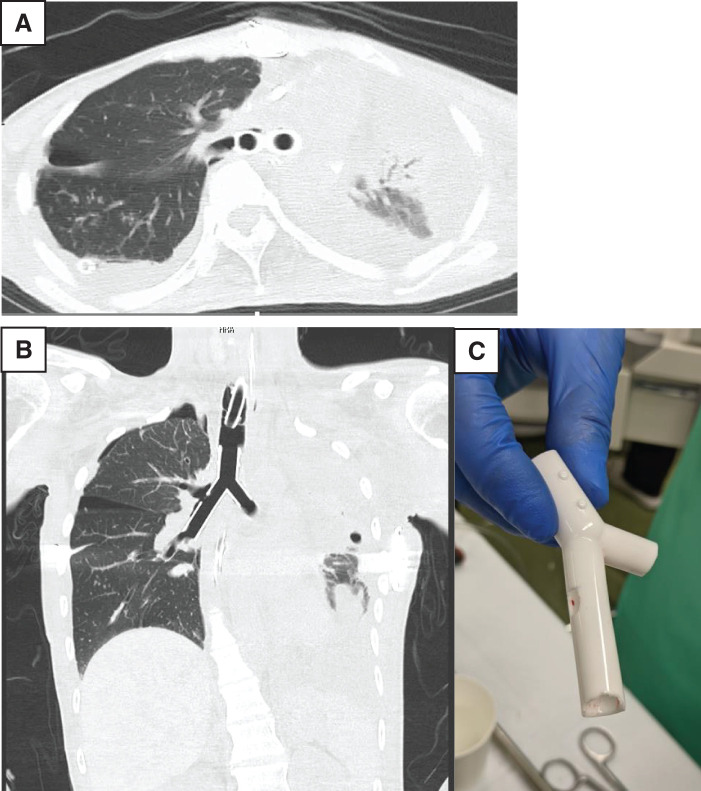
(**A**, **B**) Chest CT after stent placement: A TRACHEOBRONXANE DUMON Y-stent was implanted into the bronchial lumen without branch occlusion. (**C**) DUMON Y stent was trimmed to avoid blockage of the right upper lobe bronchus and mid-lobe bronchial inlet. TRACHEOBRONXANE DUMON Y-stent, Novatech SA, La Ciotat, France

Immediately after surgery, ventilation volume was maintained by replacement with a single-lumen tube. However, bronchial stenosis persisted during inspiration. Additionally, on the night of the surgery, short runs of AT were noted. Subsequently, the frequency of these arrhythmias, including PSVT, gradually increased. These were successfully controlled with bisoprolol fumarate (2.5 mg once daily) and verapamil (40 mg three times daily), and no further significant arrhythmias occurred. Postoperative electrocardiograms showed no significant ischemic changes. Due to the persistent inspiratory stenosis, balloon dilation of the right main bronchus was performed using bronchoscopy on POD 2 to confirm improvement in stenosis, using a 12-mm balloon (3 atm for 120 s, 1 time) and a 15-mm balloon (8 atm for 120 s, 2 times). Tracheostomy was performed on POD 3, and VV-ECMO was withdrawn on POD 4. On POD 6, a TRACHEOBRONXANE DUMON Y-stent (Novatech SA, La Ciotat, France) was placed using a rigid scope because the mediastinum was mobilized in the proper position (**Fig. 2**). The patient was transferred from the previous hospital on POD 25. After weaning from the ventilator on POD 112, the patient did not receive any oxygen therapy.

## DISCUSSION

Bronchial stenosis can be caused by various mechanisms, including congenital or acquired diseases, and benign or malignant diseases, and can present with various symptoms. Benign stenosis is often reported to be caused by postpneumonectomy syndrome, which is believed to be caused by the movement and rotation of the mediastinal organs on the affected side due to overinflation of the remaining lung and excessive absorption of pleural effusion on the affected side.^1,2)^ Similar clinical conditions have been reported, even without pneumonectomy, such as severe scoliosis^3)^ and traction on the affected side due to a collapsed lung.^4)^ To the best of our knowledge, this is the first reported case of severe bronchial disease caused by a combination of scoliosis and lung destruction caused by radiation pneumonitis.

Various treatments for benign main bronchial stenosis have been reported, including bronchoscopic balloon dilation, stent placement, the Nuss procedure, and tracheal sleeve resection.^3,5–7)^ However, there is no clear consensus regarding the choice between surgery and bronchoscopic interventions.^8)^ In the present case, bronchoscopic stenting was considered as the initial treatment; however, this treatment was deemed ineffective, as the tracheal bifurcation angle was open because of a large mediastinal deviation, and there were concerns about stent deviation and necrosis of the bronchial wall due to compression of the vertebral body. Moreover, an orthopedic anterior vertebral resection was considered but was not performed because the deformity was mild and scoliosis was not the primary cause of bronchial stenosis. Indeed, surgical intervention for scoliosis is typically considered for Cobb angles of 40°–50° in adolescents to prevent progression or >80° for respiratory dysfunction.^9)^ Our patient’s Cobb angle of 34° was below this surgical threshold, and the primary driver was the mediastinal shift from lung destruction. Furthermore, Macaré van Maurik AF et al. had noted that stent placement alone often does not correct mediastinal displacement, which is the main cause of bronchial stenosis.^10)^ One reason the Nuss procedure was ineffective in our case was that traction of the bronchus for approximately 2 years caused TBM. It is speculated that prolonged compression and traction on the bronchus induced or exacerbated TBM. Sternal elevation by the Nuss procedure alone may not have been sufficient to correct the luminal collapse due to this malacia. This contrasts with pediatric cases of severe scoliosis (e.g., Cobb angle >100°), where posterior fixation alone has been reported to successfully treat stenosis.^11)^ In adult cases with chronic compression, as in our patient, secondary TBM represents an important consideration.

Management with stenting alone is contraindicated because of the risk of migration and erosion associated with severe displacement. Therefore, lifting the mediastinal organs in the ventral direction is considered the fundamental treatment strategy. By correcting the extrinsic shift, this mobilization creates a safe environment for concomitant stenting, which was deemed essential to scaffold the airway against the intrinsic TBM.

Essaleh et al. reported a case of right main bronchial stenosis due to postpneumonectomy syndrome with a funnel chest treated with ventral sternal elevation and mediastinal mobilization with pericardial fixation to the sternum, followed by the insertion of a tissue expander into the left chest.^12)^ In the present case, the insertion of a tissue expander was not considered appropriate because the patient had not undergone a pneumonectomy. Therefore, we aimed to achieve mediastinal mobilization by pericardial suturing of the sternum and ribs.

By lifting the pericardium in various directions while observing the bronchial lumen with a bronchoscope, we sought the direction in which the lumen would be most enlarged by the pericardial suspension, and selected the ventral left side with the most luminal expansion. Regarding the suture fixation method, to prevent changes in the direction or tension of the suspension after sternal closure, we pierced nonabsorbable threads at several points through the intercostal space and fixed them after sternal closure. This procedure resulted in a stable and firm mediastinal mobilization. The pericardial suspension performed in this case aimed to effectively retract the mediastinum anteriorly and to the left by fixing the pericardium to the left anterior chest wall, thereby achieving patency of the right main bronchus. Unlike pericardial fixation to the sternum reported by Essaleh et al.,^12)^ our method allows for more extensive mediastinal mobilization and the determination of the optimal traction direction under direct bronchoscopic observation of luminal expansion. Furthermore, suturing and fixing the pericardium to the body surface after sternal closure, which prevents the reduction of the suspension effect due to chest closure and maintains stable fixation, is another crucial aspect of this technique.

In this case, the mechanism of bronchial stenosis was rightward traction and posterior compression due to scoliosis, leftward traction of the mediastinum due to lung destruction caused by radiation pneumonitis, and TBM caused by long-term compressive stenosis. Good outcomes were achieved by combining different treatment methods, namely, sternal elevation, mediastinal repositioning, and stent placement. While the combination of pericardial suspension and stent placement yielded good short-term outcomes in this case, careful long-term follow-up is necessary for potential stent-related complications (e.g., granulation tissue formation, restenosis, infection) and the possible impact of pericardial fixation on cardiac function. Although transient postoperative arrhythmias (AT and PSVT) occurred in our case, they were well-controlled pharmacologically, and no significant ischemic changes were observed. Moreover, this technique may be challenging in cases with severe adhesions or fragile tissues, highlighting the importance of careful patient selection.^13)^

## CONCLUSIONS

Major bronchial stenosis caused by mediastinal deviation may occur for reasons other than postpneumonectomy syndrome and is often difficult to treat. Various treatment options should be considered depending on the patient’s condition. Our novel mediastinal mobilization method using pericardial suspension is one of the most effective options.

## SUPPLEMENTARY MATERIALS

Supplementary Video

## DECLARATIONS

### Availability of data and materials

The datasets supporting the conclusions of this article are included within the article and its additional files.
